# The Relationship between Neighborhood Social Capital and the Health of Chinese Urban Elderly: An Analysis Based on CHARLS2018 Data

**DOI:** 10.3390/healthcare11060909

**Published:** 2023-03-21

**Authors:** Ziqi Huang, Cuihong Long, Chengzhi Yi

**Affiliations:** 1 School of Public Administration, Beihang University, Beijing 100191, China; 2 School of Economics, East China Normal University, Shanghai 200062, China; 3 School of International and Public Affairs, China Institute for Urban Governance, Shanghai Jiaotong University, Shanghai 200030, China

**Keywords:** neighborhood social capital, self-rated health, urban elderly

## Abstract

There is growing interest in the relationship between neighborhood social capital and the health of urban older people, but existing research still falls short in exploring the relationship between the two. Based on 2018 CHARLS data, this paper quantitatively examines the association between neighborhood social capital and the self-rated health of urban older people. The study found that, after controlling for a series of variables, both increased social interaction and increased frequency of social interaction significantly improved urban older people’s self-rated health. To implement the Health China strategy and improve the health of urban older people, further attention should be paid to the role of neighborhood social capital, creating a harmonious environment for neighborhood interaction and promoting the cultivation of neighborhood social capital.

## 1. Introduction

As China’s economy has continuously developed since the reform and opening up, public concern for health has been increasing. At present, China’s population structure is experiencing two significant trends: a deepening level of urbanization and an increasing aging population. With the progression of reform and opening up, urbanization in China has been rapidly advanced, leading to a significant influx of people from rural areas to cities. At the same time, the proportion of the elderly in the total population has been increasing. Data from the seventh national census bulletin show that, as of 1 November 2020, the proportion of China’s population living in urban areas was 63.89%, up 14.21 percentage points compared to data from the sixth national census in 2010. In terms of ageing, China’s population aged 60 and above reached 264 million, accounting for 18.70%, of which 191 million, or 13.50%, were aged 65 and above. Compared to the sixth national census in 2010, the proportion of the population aged 60 and over rose by 5.44 percentage points, and the proportion of the population aged 65 and over rose by 4.63 percentage points (Please refer to the Seventh National Census Bulletin (No. 1), http://www.stats.gov.cn/tjsj/tjgb/rkpcgb/qgrkpcgb/202106/t20210628_1818820.html, (accessed on 17 March 2022)).

With the reform of the market economy system, the acceleration of urbanization and the expansion of the commercialization of housing, China’s urban society has undergone rapid changes. Traditional urban communities have experienced increasing mobility of their members. New urban communities in different types of cities are being formed in large numbers. Researchers have highlighted that social capital reflects the strength of the communities in which individuals live and may have an impact on health [1]. The relationship between social capital and health has been more extensively analyzed in the existing literature at the individual or district level, but remains under-analyzed at the neighborhood level. Concerning the existing literature, researchers have analyzed the correlation between neighborhood social capital and health mainly in Western contexts, but less in non-Western contexts. For example, an empirical study based on Dutch data found a positive correlation between neighborhood social capital and health [2]. A study based on data from 239 community residents in England and Scotland did not confirm the main effect of neighborhood social capital on mental health, but found some complex associations between neighborhood social capital and mental health [3]. Neighborhood social capital might exert health effects of residents owing to their social interactions, the creation of supportive environments and the formation of community norms. There is growing interest in the correlation between neighborhood social capital and the health of older people in cities, but the relationship between the two remains under-explored by researchers.

To fill the gaps in existing research, this paper proposes to investigate the relationship between neighborhood social capital and the self-rated health of older residents based on data from the China Health and Retirement Longitudinal Study (CHARLS) 2018. The main contributions of this paper are as follows. First, this paper analyzed the relationship between neighborhood social capital and the health of urban older people. The previous literature has studied neighborhood social capital and health [2,3,4], but there remains a discrepancy. Second, this paper studied neighborhood social capital and the self-rated health of urban older residents using the latest available CHARLS 2018 data. Although the existing literature has studied the correlation between social capital and health, it has been based mainly on Western contexts, with less coverage of non-Western contexts. Third, we contribute to the literature by exploring the heterogeneity of the relationship between neighborhood social capital and health in terms of age and educational attainment. Researchers have noticed differences in the social capital of urban and rural neighborhoods and their health consequences [4], but the relationship between social capital and health in urban neighborhoods remains to be further tested.

The remainder of the paper is structured as follows: the second part introduces the literature review, analyzes the theoretical basis and hypothesis, and describes the data, variables and methods; the third part analyses the empirical results; and finally, we present the conclusion and discussion.

## 2. Materials and Methods

### 2.1. Literature Review

Health is considered to be a state of full physical, mental and social well-being [5]. Self-rated health is a widely used indicator of health. Existing studies have analyzed the correlation between social capital and individual health at the individual, community, and regional levels.

The first is the relationship between individual-level social capital and individual health. A study by Berkman et al. based on US data showed that during the follow-up period, people who lacked social and community ties had higher mortality rates than those with more extensive contact [6]. Hyyppa et al. studied the relationship between self-rated health and the social capital of residents in bilingual Swedish and Finnish-speaking communities in Finland and found that Swedish-speaking residents rated their health higher due to the fact that Swedish-speaking residents had a higher stock of social capital [7]. Using nationally representative data from Australia in 2006 to explore the relationship between structural and cognitive social capital and three types of health, general health, mental health and physical functioning—Berry et al. found that both the structural and cognitive components of social capital were associated with higher levels of health after the inclusion of control variables [8]. Considering the heterogeneity of social capital, Norstrand et al.’s study of urban–rural differences in older Chinese adults using 2005 CGSS data found that, among urban older adults, bonding social capital was positively correlated with physical and mental health, and linking social capital was correlated with physical health among urban older adults; however, no significant association between social capital and health was found among older rural adults [9]. A study conducted by Nummela et al. in Southern Finland also found that the positive relationship between self-rated health and social capital was significant in urban areas after controlling for contextual variables [10].

The second is the relationship between social capital and individual health at the community level. Subramanian et al.’s study of the relationship between social capital and health, using data from 40 community surveys in the United States in 2000, showed that high community social trust implies higher ratings of one’s own health [11]. Van Hooijdonk et al. found that residents of communities with high social capital had a lower risk of death from cancer and suicide [12]. Helliwell and Putnam used large sample data to examine the relationship between different dimensions of social capital and subjective well-being and found that communities with high levels of trust and trustworthiness improved individual health [13].

The third is the relationship between social capital and individual health at the district level. Kawachi et al.’s study showed that residents living in districts with low levels of social trust are more likely to self-assess as unhealthy [14]. An empirical study of the relationship between social capital and self-rated health in rural and urban China using CGSS2005 data by Meng and Chen found that, in a county with high bonding trust, people with high bonding trust received more health benefits than others; while in a county with low bonding trust, the opposite was true [15].

Researchers have also analyzed the mechanisms by which the link between social capital and health occurs. Kawachi et al. argues that at the national level, an individual’s political participation would have health implications, while at the community level, social capital enhances health through informal processes of social control, the maintenance of health norms and the provision of various forms of social support; at the individual level, social capital increases the likelihood of receiving various forms of social support when needed [14]. Berkman et al. propose a link between the resources of social networks and health, in which participation in social networks offers social support that may influence health by acting as a ‘buffer’ against stress. Social influence could be another route between social networks and health. In addition, social engagement offers the chances to learn new skills and gives a person a sense of belonging to a community [16]. In a review analyzing social capital as a determinant of health, Macinko and Starfield noted that existing research suggests that it is likely that high regional social capital improves regional health because of access to more government health resources [17].

From the above analysis, it can be seen that the existing literature has provided a relatively rich analysis of the relationship between social capital and individual self-rated health, providing an important basis for our understanding of the health effects of social capital. However, there are some limitations in the existing research: on the one hand, the relationship between social capital and the health of urban elderly has been under-explored in the existing literature. The elderly, as a relatively disadvantaged social group, also tend to be relatively poorer in terms of health status. In China, the rural area is traditionally a society of acquaintances, while the urban area is regarded as a society of strangers, and the degree of social capital in urban areas is generally lower than in rural ones [2]. It is worth further analyzing whether the differences in social structure between urban and rural areas bring about urban–rural differences in the link between the elderly’s social capital and health. On the other hand, the existing literature has analyzed the link between social capital and health more specifically at the individual and district levels [6,14], but the relationship between social capital and health at the neighborhood level has been under-explored, and the connection between neighborhood social capital and health for the elderly in urban China are particularly under-analyzed, which is not conducive to a better understanding of the health effects of social capital for the urban elderly.

### 2.2. Theories and Hypotheses

In the last two decades, social capital theory has emerged and evolved in academia. However, researchers have not reached a consensus on the definition of social capital. Bourdieu defined social capital as the sum of actual or potential resources, which are linked to a network of relationships that are to some extent institutionalized and available to each member of the collective [18]. From a functionalist perspective, Coleman identifies different forms of capital. He argues that social capital, although it includes many different entities, has two common elements: first, they all concern some parts of the social structure; and second, they are able to promote specific behaviors of actors within the structure [19]. According to Lin, social capital is capital acquired through social relations that could function with the resources of the network in which the actors are located [20]. In the field of political science, Fukuyama argues that social capital could be defined as an informal set of values or norms shared by group members that facilitate cooperation, and as informal norms that help individuals to cooperate with each other [21]. Putnam defines social capital as a network of social relationships formed by personal interactions, mutual benefit and reciprocity, and norms of mutual trust [22].

In the literature analysis mentioned above, although researchers analyzed the relationship between social capital and residents’ self-rated health at different levels, most of the literature supports a significant relationship between social capital and self-rated health. Focusing on the community interaction level, social capital can facilitate more rapid dissemination of health information, increase the likelihood of the adoption of health behavior norms, and exert social control over abnormal health-related behaviors [14]. Additionally, social capital is seen as a form of economic capital, a collective property that confers credit to members and is maintained and strengthened as member interactions are enhanced [20]. Therefore, residents with higher social capital are more likely to obtain resources, information, or services from social networks to enhance their health. Meanwhile, neighborhood social capital may be influenced by individual characteristics including age and education level, based upon which the following hypothesis is proposed:

**Hypothesis** **H1:** *Neighborhood social capital has a positive association with the self-rated health of urban older adults.*

At the same time, individual community interactions may be influenced by age, education level and other factors, and thus reflect individual differences, which brings about individual heterogeneity in the relationship between neighborhood social capital and the self-rated health of urban elderly. Based on this consideration, this paper proposes the following hypothesis.

**Hypothesis** **H2:** *The relationship between neighborhood social capital and self-rated health of urban older adults reflects the characteristics of individual heterogeneity.*

### 2.3. Data, Variables and Methods

#### 2.3.1. Data Source

The data used in this paper are from the China Health and Retirement Longitudinal Survey (CHARLS). The project is hosted by the National Development Research Institute of Peking University and jointly implemented by the China Social Science Survey Center of Peking University and the Peking University Youth League Committee. The aim of the survey was to collect a set of high quality micro-data representative of Chinese middle-aged and older people aged 45 and above, both at the household and individual levels. The survey sampling was divided into four stages, at the county (district), village (community), household and individual levels. Specifically, the survey used probability sampling proportional to population size in both the county (district)- and village (community-level sampling. In the village-level sampling stage, the survey followed the PPS method, based on the 2009 resident population of every village or community, and three villages or communities were randomly selected from each of the 150 districts and counties mentioned above, resulting in 450 villages/communities. The national baseline survey was conducted in 2011 and survey interviews in 150 counties and 450 communities (villages) across 28 provinces, autonomous regions and municipalities directly under the Central Government in 2011, 2013, 2015 and 2018, respectively, were obtained. The sample had covered 19,000 respondents in a total of 12,400 households in 2018 when the national follow-up survey was completed. This paper used the CHARLS2018 data (for an overview of the CHARLS survey and specific data access, please visit http://charls.pku.edu.cn/index.htm, accessed on 17 March 2022). The sample obtained after data cleaning according to the needs of the study included 6642 valid observations.

#### 2.3.2. Variable Design

##### Explained Variables

The explained variable in this study was self-rated health. Self-rated health has been widely used by scholars as a measure of health [23,24]. We chose the variable of self-rated health to measure health by referring to the empirical practices of the existing literature. A relevant question in the questionnaire asked respondents, “Would you say your health is very good, good, fair, poor or very poor?” The answer options included five levels from 1 to 5, where 1 indicates “very good” and 5 indicates “very bad”. For ease of explanation, we have reverse-coded the answer options, with 1 indicating “very bad” and 5 indicating “very good”.

##### Explanatory Variables

The core explanatory variables in this study were neighborhood social interactions and frequency of neighborhood social interactions. Different methods have been used in the literature for the measurement of social capital. Some studies use a single variable to measure social capital: e.g., Zhu et al. use social networks to define social capital [25] and Sundquist and Yang use the proportion of votes cast in elections to define neighborhood linking social capital at the community level [26]. Chetty et al. categorized social capital into connections between people across types, social cohesion, and civic engagement [1]. This paper focused on the interaction behavior of urban older adults in the community; therefore, the neighborhood social interaction in this study was measured by whether the respondents engaged in social activities such as playing ma-jong, chess, cards, and going to the community club in the past month, to which the answer option of “yes” was assigned a value of 1 while “no” was assigned a value of 0. The frequency of social interaction in the neighborhood was measured by respondents’ responses to the question, “How often did you play ma-jong, chess, cards, or go to the community club in the past month?”. The answer options included three levels from 1 to 3, where 1 indicates “almost daily”, 2 indicates “almost weekly” and 3 indicates “not regularly”. We re-coded this variable. Given that the mean value of frequency of neighborhood social interactions was 2.037, we reassigned the answer option “not regularly” or “almost weekly” to 0, indicating low frequency of neighborhood social interactions, and the answer option “almost daily” to 1, indicating high frequency of neighborhood social interactions.

##### Control Variables

Based on the empirical practice of existing studies, this study controlled for gender (male = 1, female = 0), age (the population of this paper was urban residents aged 60 and above, and was divided into three age groups: 60–69, 70–79, and 80 and above), education level (primary school and below = 1, junior high school = 2, senior high school and equivalent = 3, university and above = 4), political identity (Party membership = 1, others = 0), religious belief (yes = 1, no = 0), marital status (married = 1, others = 0), income status (including various types of pensions and benefits), household living expenses (measured by the natural logarithm of the sum of all consumption expenditures by the respondent’s household in the past year), drinking status (yes = 1, no = 0), smoking status (yes = 1, no = 0), medical insurance (yes = 1, no = 0).

**Figure 1:**
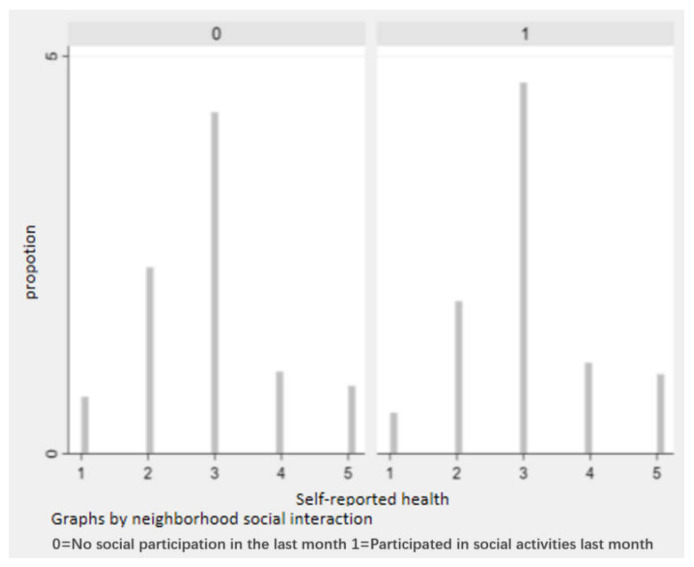
Bar distribution of self-rated health and neighborhood social interactions.

Table 1 shows the descriptive statistical results of the variables. The statistical results show that the mean value of the self-rated health score of the urban elderly in the sample is 2.910, which is slightly lower than the level of “average”. This indicates that urban elderly do not rate themselves as having high health scores. The mean value of neighborhood social interaction among urban elderly is 0.160, which indicates that the interaction among urban neighbors is generally low; while among urban elderly who have neighborhood social interaction, the frequency of interaction is more frequent. Figure 1 presents the bar distribution of the self-rated health levels of urban elderly with and without neighborhood social interaction, from which it can be seen that a larger proportion of elderly with neighborhood social interaction rated their own health more highly. This initially shows a positive relationship between neighborhood social capital and self-rated health.

However, the analysis above is a simple statistical relationship between self-rated health and the key explanatory variable obtained without controlling for other influencing factors, and the exact relationship between the two is to be verified by further analysis later.

#### 2.3.3. Methods

The explained variable of this paper, self-rated health, is an ordered categorical variable including five different levels. Different scholars have different views on whether an OLS model or an ordered logistic (Ologit) model should be used for ordered categorical variables. To be on the safe side, this paper drew on empirical practice and used both OLS and Ologit models for estimation [27]. Additionally, this paper tested the age and educational differences of neighborhood social capital in terms of the self-rated health of urban elderly. To help overcome possible estimation bias due to self-selection by referring to existing literature [27,28], this paper used the propensity score matching (PSM) method to further identify the relationship between the social capital and self-rated health of urban older adults. Before using the Ologit model, this paper conducted a multicollinearity test. Specifically, we tested whether there was a multicollinearity problem between explanatory variables. The condition number obtained by running the coldiag2 [29] command was 23.54, which is smaller than the empirical standard value of 30, so it could be considered that there is no serious multicollinearity problem.

## 3. Results

### 3.1. Analysis of the Association of Neighborhood Social Capital with Self-Rated Health of Urban Older Adults

Table 2 shows the results for estimating the OLS and Ologit models of the relationship between neighborhood social interaction or frequency of neighborhood social interaction and the self-rated health of urban older adults. Models 1 and 2 were estimated using the OLS model. In this paper, we first considered the association between neighborhood social interactions and the self-rated health of older adults in urban areas, followed by the association between the frequency of neighborhood social interactions and the self-rated health of older adults in urban areas. Models 3 and 4 were then estimated using Ologit models for the above models. The estimation results of Model 2 showed that social interaction had a significant positive relationship with urban older adults’ self-rated health after controlling for a range of relevant variables; urban older adults with social interaction rated their health higher compared to those without social interaction among urban elderly; one possible reason is that, in recent years, China has taken many initiatives to enhance urban neighborhood cohesion, which has deepened the level of interaction among residents, increased the social capital of urban neighborhoods, and thus promoted urban residents’ self-rated health. Model 2 showed that the frequency of social interaction was significantly and positively associated with urban older adults’ self-rated health. Urban older adults with high frequency of social interaction rated their health more highly than urban older adults with low frequency of social interaction. Models 3 and 4 were estimated using the Ologit model, and the estimation results were generally consistent with those estimated using the OLS model.

### 3.2. Discussion on the Issue of Self-Selection

The estimation of the relationship between neighborhood social capital and the health of urban older people could suffer from the issue of self-selection. For example, more healthy individuals may choose to move into or live in communities with higher neighborhood social capital, and these residents may generate higher social capital to the surrounding environment, while the surrounding environment could further attract a certain kind of individuals, thus individuals may not be randomly allocated across neighborhoods (we thank the anonymous reviewer for clearly stressing the issue of self-selection). To address the concerns of self-selection and more accurately measure the relationship between neighborhood social capital and the health of urban older people, we adopted the method of propensity score matching (PSM). When allocating matching scores, various methods, including one-to-one nearest neighbor matching, nearest neighbor matching within caliper and kernel matching were used in the analysis. The application of the PSM method is subject to the balance test of matching variables.

Table 3 reports the average treatment effect on the treated (ATT), with neighborhood social interaction as the treatment variable. The estimation results show that, after matching, respondents who have neighborhood social interaction (treatment group) tend to have higher self-rated health than those who have no neighborhood social interaction (control group). The difference is approximately 9.4% to 16.9%. Individuals with a high frequency of social interaction in the neighborhood (treatment group) have higher self-rated health than those with a low frequency of social interaction in the neighborhood (control group). The difference is approximately 16.9% to 19.4%. The differences between the treatment group and the control group after propensity score matching with the use of different matching methods are all statistically significant. In conclusion, there is a significant positive association between the neighborhood social interaction and the health of urban older people, and also a significant positive association between the frequency of social interaction in the neighborhood and the health of urban older people. The application of propensity score matching requires the balance of each confounding variable. Table A1 and Table A2 in Appendix A present the standardized bias and t-values before and after matching. The results show that the standardized bias between the treatment and control groups was larger before matching than after matching. Specifically speaking, after matching, the standardized bias was less than 10% for every confounding variable, indicating that there is no systematic difference between the confounding variables of the treatment and control groups after propensity score matching. After matching, *t*-tests for each confounding variable between the treatment and control groups were no longer significant. This implies that the covariates in the treatment and control groups are balanced [30]. Thus, the PSM analysis results remain consistent with those of the ordinary logit and OLS estimation, further confirming the robustness of the results.

### 3.3. Interaction Analysis

#### 3.3.1. The Interaction Analysis between Neighborhood Social Capital and Age on Health

The previous analysis confirmed that neighborhood social capital has a significant positive association with the self-rated health of urban older adults. Considering the increasing educational attainment of Chinese residents and the ageing of society in recent years, we further tested whether the relationship between neighborhood social capital and the health of urban older people reflects age and education heterogeneity. In the following analysis, urban older adults were divided into the two groups of 60–69 years old and 70 years old and above based on age, and two groups with higher and lower education levels using the mean value of education level as the splitting point.

Models 4a and 4b in Table 4 reported the coefficients of the interaction term between social interaction and age, as well as the interaction term between the frequency of social interaction and age, respectively. Among them, only the coefficient of the interaction term between the frequency of social interactions and age is significant. Thus, Hypothesis H2 was partially confirmed here.

To make it easier to understand the association of change in the explanatory variable with the predicted variable, the coefficients presented in the model estimates are odds ratios. The results showed that the coefficient of the interaction term between the frequency of social interaction and age was significantly positive, which indicated that the frequency of social interaction played a more prominent role in respondents’ self-rated health as their age increased. A possible reason for this result is that urban older adults aged 70 years and older are generally in poorer physical condition, have a more limited range of activities, and thus could be more dependent on social interactions within the community for their health benefits.

#### 3.3.2. The Interaction Analysis between Neighborhood Social Capital and Education Level on Health

Models 4c and 4d in Table 4 reported the coefficients of the interaction term between social interaction and education level, as well as the interaction term between the frequency of social interaction and education level, respectively. Among them, only the interaction term between the frequency of social interactions and education level is significant.

**Table 4:** Results of interaction analysis.

	Model 4a	Model 4b	Model 4c	Model 4d
	Self-Rated Health	Self-Rated Health	Self-Rated Health	Self-Rated Health
Social interaction	1.178 ***		1.169 **	
	(0.074)		(0.076)	
Frequency of neighborhood social interaction		1.444 ***		1.480 ***
		(0.180)		(0.195)
Age ^a^	0.858 ***	0.936	0.858 ***	0.936
	(0.043)	(0.119)	(0.043)	(0.120)
Gender	0.998	0.892	0.997	0.892
	(0.061)	(0.137)	(0.061)	(0.137)
Education level ^b^	1.428 ***	1.535 ***	1.428 ***	1.507 ***
	(0.088)	(0.202)	(0.089)	(0.198)
Political identity	1.146 *	1.226	1.147 *	1.238
	(0.085)	(0.190)	(0.085)	(0.192)
Religious beliefs	1.139 *	1.140	1.140 *	1.185
	(0.088)	(0.316)	(0.088)	(0.330)
Marital status	1.122 **	0.993	1.124 **	0.980
	(0.062)	(0.140)	(0.062)	(0.139)
Income status	1.000 ***	1.000	1.000 ***	1.000
	(0.000)	(0.000)	(0.000)	(0.000)
Household living expenses	0.904 ***	0.910 ***	0.904 ***	0.910 ***
	(0.010)	(0.027)	(0.010)	(0.027)
Alcohol consumption status	1.494 ***	1.317 **	1.494 ***	1.314 **
	(0.083)	(0.174)	(0.084)	(0.174)
Smoking status	1.058	1.335 **	1.058	1.308 *
	(0.064)	(0.186)	(0.064)	(0.183)
Medical Insurance	0.918	1.111	0.919	1.144
	(0.126)	(0.488)	(0.126)	(0.523)
Interact term				
Social interaction × Age	1.193			
	(0.150)			
Frequency of neighborhood social interaction × Age		1.970 ***		
		(0.489)		
Social interaction × Education level			0.960	
			(0.118)	
Frequency of neighborhood social interaction × Education level				0.634 *
				(0.153)
/cut1	0.063 ***	0.074 ***	0.063 ***	0.072 ***
	(0.011)	(0.041)	(0.011)	(0.041)
/cut2	0.387 ***	0.467	0.389 ***	0.457
	(0.067)	(0.251)	(0.068)	(0.254)
/cut3	3.378 ***	4.776 ***	3.396 ***	4.633 ***
	(0.590)	(2.570)	(0.595)	(2.577)
/cut4	8.628 ***	12.119 ***	8.671 ***	11.748 ***
	(1.526)	(6.569)	(1.538)	(6.574)
Observations	6250	1074	6250	1074

Note: Coefficients reported are odds ratios; Robust standard errors in brackets, * *p* < 0.1, ** *p* < 0.05, *** *p* < 0.01. The reference groups for the categorical variables are: ^a^ 60–69 years old, ^b^ Primary school and below.

The results showed that the coefficient of the interaction term between the social interaction frequency and educational attainment was significantly negative, indicating that for the group with below-average educational attainment, an increase in the frequency of social interactions had a more significant effect on respondents’ self-rated health. It might be that neighborhood social capital has a more complementary association with the health dimension for urban older adults with a lower than the average level of education.

## 4. Discussion and Conclusions

Previous studies have focused more on the impact of social capital at the level of the individual or larger geographic unit [6,7,8,9,10,14,15,31,32,33]. However, individuals are likely to be influenced by the communities in which they live their daily lives. Therefore, unlike previous studies [34,35], this paper was based on the analysis of representative data from China and focused on the neighborhood level. Using data from CHARLS2018, we empirically investigated the relationship between neighborhood social capital and the self-rated health of urban older adults. It was found that both social interaction and the frequency of social interaction have a significantly positive association with urban older adults’ self-rated health after controlling for a range of variables, which is consistent with the findings of previous studies [2].

With rapid urbanization and ageing, more and more younger Chinese are moving from the countryside to work and live in the city. A considerable number of young people prefer to pursue their own lifestyles and tend not to live with their parents, which has led to an increasing number of elderly people living alone [36]. This could affect the health of urban elderly. In addition, older people are already at a relative disadvantage in terms of their health status compared to other age groups. This paper confirms a significant positive relationship between neighborhood social capital and the health of urban older people. The existing literature has analyzed the possible mechanisms of the relationship. A study suggests that neighborhood social capital may contribute to the health of residents by enhancing the ability of communities to provide services [2]. However, the mechanisms of the relationship between neighborhood social capital and the health of urban older people remain to be explored.

Further heterogeneity analysis revealed that the frequency of social interaction had a significant correlation with both the self-rated health of urban elderly aged 70 years or above and lower education level. It has been shown that the proportion of older adults experiencing social isolation increases with age and that older adults with low levels of education are at high risk of social isolation [37]. As a psychological resource, social participation can improve an individual’s self-evaluation of health, which could become more pronounced with age [38].

The above findings have important policy value. Considering that self-rated health is seen as a valid measure of health and the significant positive association of neighborhood social capital with urban elderly people’s self-rated health, it is necessary to further emphasize the role of neighborhood social capital, create a harmonious environment of neighborhood interaction, and promote the cultivation of neighborhood social capital to promote the Healthy China Strategy and improve the health of urban elderly people.

There are also some limitations in this paper. First, in terms of the measurement of neighborhood social capital, this paper focused on offline social capital, but with the increasing number of Internet users, the possible influence of online social capital on the lives, behavior and health of urban elderly also deserves attention, and future studies can pay more attention to the influence of online social capital on the health status of urban elderly. Second, due to the limitation of cross-sectional data and potential endogeneity issues as a result of possible omitted variables and the interplay between individual self-rated health and social interactions as well as frequency of interactions, it is actually difficult to verify the causal relationship between neighborhood social capital and self-rated health of urban older adults in this study. Future studies need to collect longitudinal data to explore this in more detail, and sufficient attention needs to be paid to the treatment of endogeneity issues in order to obtain more robust and reliable research findings.

## Figures and Tables

**Table 1:** Descriptive statistics of variables.

	Variable	Sample Size	Mean	Standard Deviation	Min	Max
Explained variable	Self-reported health	6251	2.910	1.010	1	5
Explanatory variable	Neighborhood social interaction	6642	0.160	0.370	0	1
	Frequency of social interaction in the neighborhood	1089	0.360	0.480	0	1
Control variable	Gender	6642	0.500	0.500	0	1
	Age	6642	1.580	0.710	1	3
	Education level	6642	1.360	0.700	1	4
	Political identity	6642	0.120	0.330	0	1
	Religious beliefs	6642	0.120	0.320	0	1
	Marital status	6642	0.660	0.480	0	1
	Income status	6642	8712	19,215	0	600,000
	Household living expenses	6642	8.040	2.450	0	14.510
	Smoking status	6642	0.270	0.440	0	1
	Alcohol consumption status	6641	0.310	0.460	0	1
	Medical Insurance	6642	0.970	0.180	0	1

Source: CHARLS2018.

**Table 2:** The OLS and Ologit estimation results.

Variables	Model 1	Model 2	Model 3	Model 4
	OLS	OLS	Ologit (Odds Ratio)	Ologit (Odds Ratio)
Social interaction	0.083 **		1.167 **	
	(0.034)		(0.072)	
Frequency of neighborhood social interaction		0.172 ***		1.380 ***
		(0.064)		(0.171)
Age ^a^				
70 years old–79 years old	−0.081 ***	−0.048	0.841 ***	0.893
	(0.029)	(0.070)	(0.045)	(0.122)
80 years old and above	−0.040	0.185	0.927	1.338
	(0.045)	(0.121)	(0.079)	(0.307)
Gender	0.009	−0.038	0.996	0.885
	(0.033)	(0.079)	(0.061)	(0.136)
Education level ^b^				
Junior high school	0.185 ***	0.223 ***	1.454 ***	1.636 ***
	(0.037)	(0.077)	(0.098)	(0.237)
Senior high school and equivalent	0.156 ***	0.144	1.367 ***	1.405 *
	(0.048)	(0.091)	(0.125)	(0.255)
University and above	0.223 **	−0.044	1.535 **	1.055
	(0.099)	(0.220)	(0.280)	(0.466)
Political identity	0.072 *	0.123	1.144 *	1.258
	(0.040)	(0.082)	(0.085)	(0.196)
Religious beliefs	0.073 *	0.077	1.139 *	1.142
	(0.041)	(0.135)	(0.088)	(0.320)
Marital status	0.073 **	0.024	1.134 **	1.024
	(0.030)	(0.074)	(0.063)	(0.146)
Income status	0.000 ***	0.000	1.000 ***	1.000
	(0.000)	(0.000)	(0.000)	(0.000)
Household living expenses	−0.052 ***	−0.047 ***	0.905 ***	0.912 ***
	(0.006)	(0.015)	(0.010)	(0.027)
Alcohol consumption status	0.202 ***	0.118 *	1.496 ***	1.302 **
	(0.030)	(0.068)	(0.084)	(0.172)
Smoking status	0.024	0.140 *	1.060	1.302 *
	(0.033)	(0.073)	(0.064)	(0.184)
Medical Insurance	−0.057	0.146	0.923	1.195
	(0.073)	(0.225)	(0.126)	(0.527)
Constants	3.187 ***	2.994 ***		
	(0.085)	(0.263)		
/cut1	0.045 ***	0.051 ***	0.045 ***	0.051 ***
	(0.007)	(0.028)	(0.007)	(0.028)
/cut2	0.277 ***	0.324 **	0.277 ***	0.324 **
	(0.045)	(0.171)	(0.045)	(0.171)
/cut3	2.417 ***	3.279 **	2.417 ***	3.279 **
	(0.390)	(1.720)	(0.390)	(1.720)
/cut4	6.172 ***	8.317 ***	6.172 ***	8.317 ***
	(1.010)	(4.382)	(1.010)	(4.382)
N	6250	1074	6250	1074
R2	0.041	0.044		

Note: * *p* < 0.1, ** *p* < 0.05, *** *p* < 0.01. The reference groups for the categorical variables are: ^a^ 60–69 years old, ^b^ Primary school and below.

**Table 3:** Propensity score matching results.

Variable	Matching Method	Number of the Matched Sample	Sample	Treated	Controls	Difference	SE	T-Stat
Neighborhood social interaction	One-by-one Nearest Neighbor	6236	Unmatched	3.020	2.888	0.132	0.034	3.90 ***
			ATT	3.020	2.851	0.169	0.046	3.66 ***
	Nearest Neighbor Matching within Caliper	6229	Unmatched	3.020	2.888	0.132	0.034	3.90 ***
			ATT	3.020	2.927	0.102	0.038	2.67 ***
	Kernel Matching	6235	Unmatched	3.020	2.888	0.132	0.034	3.90 ***
			ATT	3.020	2.917	0.094	0.034	2.74 ***
Frequency of Social interaction in the neighborhood	One-by-one Nearest Neighbor	1069	Unmatched	3.118	2.965	0.153	0.063	2.43 **
			ATT	3.118	2.923	0.194	0.089	2.19 **
	Nearest Neighbor Matching within Caliper	1060	Unmatched	3.118	2.965	0.153	0.639	2.43 **
			ATT	3.120	2.930	0.190	0.725	2.62 ***
	Kernel Matching	1065	Unmatched	3.118	2.965	0.153	0.063	2.43 **
			ATT	3.116	2.948	0.169	0.065	2.59 ***

Notes: ** t < 1.96; *** t < 2.58 (two-tailed).

## Data Availability

The data can be accessed by request through http://charls.pku.edu.cn/index.htm, (accessed on 17 March 2022).

## Appendix A

**Table A1:** Balance Test with Frequency of Neighborhood Social Interaction as Treatment.

	Unmatched		One-by-One Nearest Neighbor		Nearest Neighbor Matching within Caliper		Kernel Matching	
Variable	%bias	t	%bias	t	%bias	t	%bias	t
70–79 years old	12.9	2.05 **	7.2	1.000	5.4	0.730	1.1	0.150
80 years old and above	21.0	3.48 ***	−0.900	−0.110	−4.900	−0.620	1.5	0.190
Gender	−3.8	−0.6	3.7	0.510	−5.500	−0.760	−1.400	−0.200
Junior high school	−10.7	−1.67 *	−4.400	−0.630	−1.400	−0.200	−0.200	−0.020
Senior high school and equivalent	−7.0	−1.09	−8.200	−1.140	−2.000	−0.280	0.4	0.060
University and above	2.8	0.44	0	0.000	0.8	0.100	−1.100	−0.140
Political identity	4.1	0.66	−3.400	−0.450	2.9	0.410	1.7	0.240
Religious beliefs	−7.0	−1.08	6.8	1.070	2.2	0.330	0.9	0.130
Marital Status	−27.8	−4.47 ***	−0.600	−0.080	−4.600	−0.610	−3.000	−0.400
Income status	3.2	0.51	−2.000	−0.260	−9.800	−1.000	−2.000	−0.240
Household living expenses	−1.7	−0.27	−8.300	−1.190	−1.900	−0.260	0.5	0.070
Alcohol consumption status	−10.2	−1.61	−0.500	−0.070	−5.700	−0.790	−2.000	−0.280
Smoking status	−14.7	−2.31 **	6.4	0.930	3.5	0.500	−1.600	−0.220
Medical Insurance	−0.7	−0.11	3.4	0.450	4	0.510	1.2	0.160

Notes: * t < 1.65; ** t < 1.96; *** t < 2.58 (two-tailed).

**Table A2:** Balance Test with Social Interaction as Treatment.

	Unmatched		One-by-One Nearest Neighbor		Caliper Matching		Kernel Matching	
Variable	%bias	t	%bias	t	%bias	t	%bias	t
70–79 years old	−5.80	−1.70 *	4.6	1.090	2.600	0.620	−1.100	−0.260
80 years old and above	−11.80	−3.35 ***	−0.300	−0.080	2.800	0.720	0.800	0.200
Gender	25.90	7.67 ***	−0.800	−0.180	−4.100	−0.980	2.400	0.550
Junior high school	18.400	5.77 ***	−0.500	−0.100	0.600	0.140	2.400	0.530
Senior high school and equivalent	21.9	7.21 ***	−1.200	−0.250	−1.900	−0.380	3.300	0.680
University and above	4.3	1.34	−0.700	−0.150	−1.600	−0.330	−0.100	−0.020
Political identity	17.2	5.45 ***	2.9	0.630	0.200	0.050	2.900	0.630
Religious beliefs	−14.7	−4.11 ***	3.1	0.830	0.000	−0.010	−0.100	−0.020
Marital Status	14.3	4.18 ***	3.1	0.730	−2.800	−0.670	0.500	0.110
Income status	25.4	8.49 ***	6	1.210	4.200	1.150	7.200	1.460
Household living expenses	23.3	6.60 ***	0	0.000	1.900	0.490	0.500	0.140
Alcohol consumption status	23.6	7.21 ***	−0.400	−0.090	−0.700	−0.160	2.600	0.590
Smoking status	24.6	7.59 ***	−0.800	−0.180	−0.500	−0.100	3.200	0.710
Medical Insurance	8	2.22 **	−1.700	−0.450	−1.400	−0.370	0	0.000

Notes: * t < 1.65; ** t < 1.96; *** t < 2.58 (two-tailed).
